# *Pichia pastoris* Mediated Digestion of Water-Soluble Polysaccharides from Cress Seed Mucilage Produces Potent Antidiabetic Oligosaccharides

**DOI:** 10.3390/ph17060704

**Published:** 2024-05-29

**Authors:** Imdad Ullah Khan, Yusra Jamil, Aiman Khan, Jalwa Ahmad, Amjad Iqbal, Sajid Ali, Muhammad Hamayun, Anwar Hussain, Abdulwahed Fahad Alrefaei, Mikhlid H. Almutairi, Ayaz Ahmad

**Affiliations:** 1 Department of Biotechnology, Abdul Wali Khan University Mardan, Mardan 23200, Pakistan; ik16092@gmail.com (I.U.K.); yusrajamil22@gmail.com (Y.J.); khanaiman584@gmail.com (A.K.); jalwaahmad055@gmail.com (J.A.); 2 Department of Food Science and Technology, Abdul Wali Khan University, Mardan 23200, Pakistan; 3 Department of Horticulture and Life Science, Yeungnam University, Gyeongsan 38541, Republic of Korea; 4 Department of Botany, Abdul Wali Khan University Mardan, Mardan 23200, Pakistan; hamayun@awkum.edu.pk (M.H.); drhussain@awkum.edu.pk (A.H.); 5 Department of Zoology, College of Science, King Saud University, Riyadh 11451, Saudi Arabia; afrefaei@ksu.edu.sa (A.F.A.); malmutari@ksu.edu.sa (M.H.A.)

**Keywords:** diabetes mellitus, oligosaccharides, water-soluble polysaccharides, microbial digestion, *Pichia pastoris*, gel permeation chromatography, STZ-induced diabetic mice, blood glucose level

## Abstract

Diabetes mellitus is a heterogeneous metabolic disorder that poses significant health and economic challenges across the globe. Polysaccharides, found abundantly in edible plants, hold promise for managing diabetes by reducing blood glucose levels (BGL) and insulin resistance. However, most of these polysaccharides cannot be digested or absorbed directly by the human body. Here we report the production of antidiabetic oligosaccharides from cress seed mucilage polysaccharides using yeast fermentation. The water-soluble polysaccharides extracted from cress seed mucilage were precipitated using 75% ethanol and fermented with *Pichia pastoris* for different time intervals. The digested saccharides were fractionated through gel permeation chromatography using a Bio Gel P-10 column. Structural analysis of the oligosaccharide fractions revealed the presence of galacturonic acid, rhamnose, glucuronic acid, glucose and arabinose. Oligosaccharide fractions exhibited the potential to inhibit α-amylase and α-glucosidase enzymes in a dose-dependent manner in vitro. The fraction DF73 exhibited strong inhibitory activity against α-amylase with IC_50_ values of 38.2 ± 1.12 µg/mL, compared to the positive control, acarbose, having an IC_50_ value of 29.18 ± 1.76 µg/mL. Similarly, DF72 and DF73 showed the highest inhibition of α-glucosidase, with IC_50_ values of 9.26 ± 2.68 and 50.47 ± 5.18 µg/mL, respectively. In in vivo assays in streptozotocin (STZ)-induced diabetic mice, these oligosaccharides significantly reduced BGL and improved lipid profiles compared to the reference drug metformin. Histopathological observations of mouse livers indicated the cytoprotective effects of these sugars. Taken together, our results suggest that oligosaccharides produced through microbial digestion of polysaccharides extracted from cress seed mucilage have the potential to reduce blood glucose levels, possibly through inhibition of carbohydrate-digesting enzymes and regulation of the various signaling pathways.

## 1. Introduction

Diabetes mellitus is a chronic metabolic disorder characterized by high blood glucose levels resulting from defects in insulin secretion, insulin action, or both [1]. Approximately 25% of the global population is affected by diabetes [2]. It ranks as the fifth leading cause of global mortality, and its prevalence is expected to rise in the future. Current therapeutic strategies for the treatment of diabetes are mainly focused on lifestyle modification and pharmacological interventions. Several oral hypoglycemic drugs, such as iminosugars, sugar derivatives, thiazolidinediones, biguanides, α-amylase and α-glucosidase have been employed to mitigate hyperglycemia levels [3,4]. However, the limited comprehension of diabetes pathogenesis during the development of previous clinical agents has led to undesirable toxic and adverse effects, including hypoglycemia, flatulence and damage to the islet cells [5]. Consequently, there is a growing interest in exploring plant based alternatives as hypoglycemic agents to prevent this debilitating disease [6].

Plants have been used in traditional medicine for centuries to treat various ailments, including diabetes. In recent years, there has been growing interest in identifying natural plant-derived compounds as potential antidiabetic agents. Many plant species have been shown to possess antidiabetic properties in preclinical and clinical studies [7]. Plant-derived compounds can act on multiple pathways involved in glucose metabolism, such as insulin secretion, insulin sensitivity and glucose uptake [8]. Polysaccharides, composed of many monosaccharide units, are carbohydrates that have been recently shown to exhibit potential antidiabetic activity by regulating hyperglycemia. The antidiabetic potential of β-d-(1 → 6)-glucan has been reported to enhance insulin levels and promote the accumulation of hepatic glycogen, while decreasing blood glucose levels in streptozotocin (STZ)-induced diabetic mice [9]. Many of the polysaccharides from plant sources are non-digestible due to the lack of appropriate digestive enzymes in humans; however, they interact with human gut probiotics, fostering their growth and thereby promoting human health [10,11]. Some seeds of the flowering plants, when soaked in water, release a white gelatinous layer known as mucilage, which is hydrophilic in nature. Due to its variable chemical composition, mucilage has been found to exhibit diverse functional properties and have a preventive effect on many human ailments [12].

*Lepidium sativum* (*L. sativum*) belongs to the family *Brassicaceae* and is commonly known as cress, garden cress, pepperwort and peppergrass. The cress plant is extensively cultivated in the United States, the Middle East and Europe [13]. Extracts from cress have been extensively studied for various pharmacological properties, including hypoglycemic, hypotensive, antimicrobial, bronchodilator and allelopathic activities [14]. Cress mucilage derived from their seeds offers numerous pharmaceutical and therapeutic applications, along with its potential as a food additive. Being hydrocolloid in nature, cress mucilage showed promising antimicrobial and antioxidant properties. Notably, cress mucilage is cost-effective and widely employed in home-made remedies, which further enhances its versatility and utility [15]. Polysaccharides are the main constituents of cress seed mucilage that have a diverse monosaccharide composition, including mannose, galacturonic acid, fructose, glucuronic acid, arabinose, rhamnose, glucose and galactose [16]. Rich in polysaccharides, cress seed mucilage possesses unique physiochemical properties and bioactivities that make it an intriguing candidate for exploring antidiabetic potential. 

The current study aimed to study the effect of yeast on the production of antidiabetic oligosaccharides from cress seed mucilage polysaccharides through a one-step fermentation process. By evaluating the antidiabetic efficacy of these bioactive oligosaccharides and deciphering the possible mechanism of action, the findings of this study contribute to the growing body of knowledge on natural antidiabetic agents. Furthermore, understanding the bio-catalytic function of yeast cells for the effective production of antidiabetic oligosaccharides and their impact on glucose homeostasis may pave the path for the development of alternative natural and cost-effective therapeutic options for the management of diabetes.

## 2. Results and Discussion

### 2.1. Microbial Digestion of Polysaccharides Obtained from Cress Seed Mucilage

**Figure 1:**
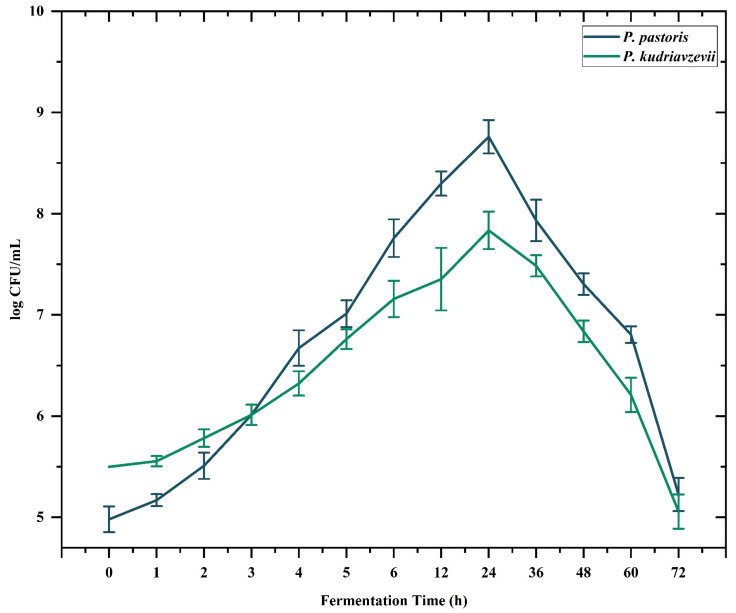
Proliferation of *Pichia pastoris* and *Pichia kudriazveii* on media containing EPP as a carbon source.

Polysaccharides are high-molecular-weight polymers that are found naturally in edible plants; however, most of them cannot be digested or absorbed directly by the human body. The biological function of polysaccharides is influenced by multiple factors, including their molecular weight, monosaccharide composition and structure [17]. Attempts have been made to modify polysaccharides for the production of more useful compounds. Oligosaccharides obtained from lignin, hemicellulose, pectin, chitin and other polysaccharides possess enormous potential [18]. The bioactive potential of oligosaccharides increases their applications in various therapeutic approaches, including improvement of gut health, immune boosting, cancer treatment and anti-adhesive action [19]. *Pichia pastoris* (*P. pastoris*), a methylotrophic yeast and a natural inhabitant of trees, demonstrates a remarkable potential for utilizing various carbon sources, including glucose, glycerol and even methanol. That is why it has gained significant attraction as an effective host for the production of several commercial products, including recombinant proteins and enzymes such as trypsin and proteinase K, phospholipase C and phytase [20]. In this study, we carried out the digestion of polysaccharides isolated from cress seed mucilage in the presence of *P. pastoris* and *Pichia kudriavzevii* (*P. kudriavzevii*). The results illustrated in Figure 1 show that significant proliferation was recorded in the growth of both yeast strains in media supplemented with EPP as compared to media having no carbon source. The maximum growth for both experimental strains was recorded after 24 h of inoculation, where the growth of *P. pastoris* increased by 4.98 ± 0.13 log CFU/mL of the initial count to 8.76 ± 0.17 log CFU/mL after 24 h and that of *P. kudriavzevii* was increased to 7.84 ± 0.19 log CFU/mL from 5.50 ± 0.00 log CFU/mL. It has been reported previously that fermentation of rice brain polysaccharides (RBPSs) by the fungus *Grifola frondosa* yields bioactive oligosaccharides that show promising antioxidant potential against DPPH free radicals along with regulating nitric oxide (NO) production [21]. 

### 2.2. Total Carbohydrates Content of the GPC Fractionated Saccharides

**Figure 2:**
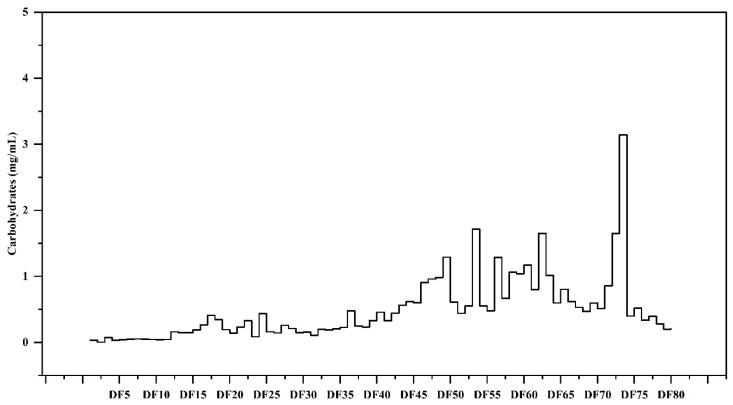
Total carbohydrates in oligosaccharide fractions obtained through microbial digestion of EPP.

The phenol–sulfuric acid (PSA) method is one of the most commonly applied and reliable methods for the quantification of total sugar contents. The principle of the PSA method is that H_2_SO_4_ dehydrates the polysaccharides to uronic acid and hydroxyurea formaldehydes. The hydroxyurea formaldehydes produced then react with phenol, resulting in the production of an orange-red or deep orange color, which can be quantified by measuring the absorbance at 490 nm. The concentration of the sugar present is directly proportional to the intensity of the color [22]. The total carbohydrate content (TCC) of EPP digested with *P. pastoris* and fractionated with GPC is illustrated in Figure 2. Among the collected fractions, fraction DF73 contains 3.14 ± 0.66 mg/mL of the TCC. The results show that *P. pastoris* effectively digested the EPP, as no high-molecular-weight polysaccharides were observed in fractions DF1 to DF29, while a very low quantity of carbohydrates was observed in fractions DF30 to DF45. DF53 contains 1.71 ± 0.32 mg/mL of TCC, which was followed by DF72 (1.65 ± 0.27 mg/mL), DF49 (1.29 ± 0.91 mg/mL), DF56 (1.19 ± 0.69 mg/mL) and DF60 (1.17 ± 0.83 mg/mL). Fractions with a considerable amount of carbohydrates were further assayed for their biochemical and biological potential.

### 2.3. Monosaccharides and Disaccharides Composition of Oligosaccharide Fractions

The monosaccharide composition of selected oligosaccharide fractions was examined using GC-MS, which confirms the presence of neutral and acidic sugars. The results depicted in Table 1 describe that DF53, DF72 and DF73 contain various concentrations of fucose, arabinose, galactose, glucose, glucuronic and galacturonic acid. These results suggest that the monosaccharide composition of DF53, DF72 and DF73 was almost the same, where galacturonic acid is the most abundant acidic monosaccharide. The high percentage of galacturonic acid coincides with the results of FT-IR, where a broad band was obtained for the presence of uronic acids. Literature shows that mucilage obtained from *Corchorus olitorius* has the highest uronic acid content, followed by galactose, rhamnose and arabinose [23]. The monosaccharide composition of polysaccharides extracted from *Opuntia ficus-indica* gum confirms the presence of the same monosaccharide units as reported in our study but with different concentrations [24]. Similarly, polysaccharides extracted from *Lepidium perfoliatum* seed mucilage were shown to contain rhamnose, galactose, arabinose, glucose and xylose in different quantities [25]. 

### 2.4. IR Spectra of Oligosaccharide Fractions

**Figure 3:**
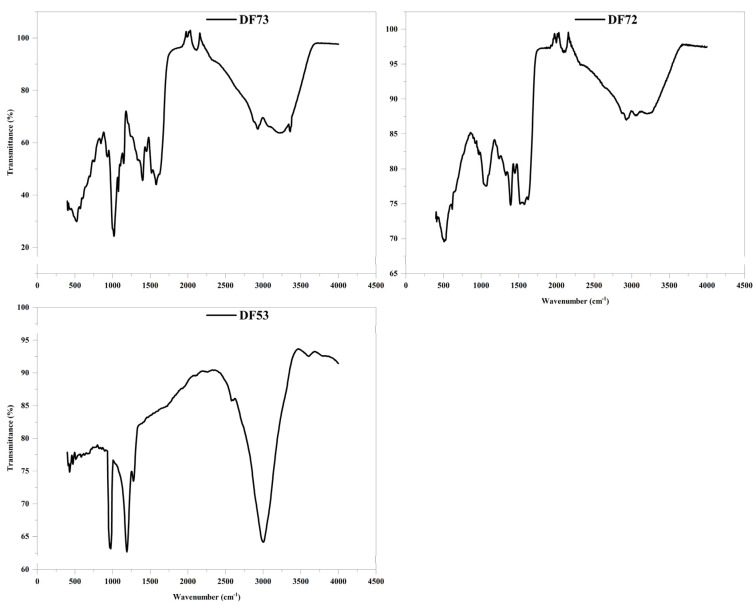
IR–spectroscopy of the DF53, DF72 and DF73 oligosaccharide fractions.

Fourier-transform infrared (FT-IR) spectroscopy is an effective technique used for qualitative identification of specific organic groups such as –OH, N–H and C–O within bioactive macromolecules. The FT-IR spectrum provides useful information about the main functional groups of plant-derived polysaccharides [26,27]. The FT-IR spectrum of the oligosaccharide fractions, including DF53, DF72 and DF73, obtained through microbial digestion of EPP is illustrated in Figure 3. Upon examination, a strong band at 3354 cm^−1^ was observed, which is attributed to the presence of stretching vibration of the –OH group [28]. Besides this, several other prominent bands were identified, each providing a valuable insight into the molecular structure of oligosaccharide fractions. The presence of band peaks in the range 1000 to 1200 cm^−1^ is associated with C–OH and C–O–C bonds, which are characteristic functional groups of carbohydrates and consistent with the presence of glycosidic linkages and saccharide moieties [29]. Additionally, a characteristic band was observed at 2929 cm^−1^ in DF73, which is assigned to –CH group. Particularly, an intense absorption band was observed at 1657 cm^−1^ which is commonly associated with the presence of the uronic acid (C–O–) functional group, which supported the results of the uronic acid assay and element analysis [30]. Further, the presence of peaks between 950 cm^−1^ to 970 cm^−1^ is associated with β-type glycosidic linkages. The characteristic absorption at 950 cm^−1^ to 970 cm^−1^ indicated the presence of β-glycosidic linkages between the sugar units in carbohydrates [31]. 

### 2.5. In Vitro Alpha-Amylase Inhibition Activity of the Fractions

**Figure 4:**
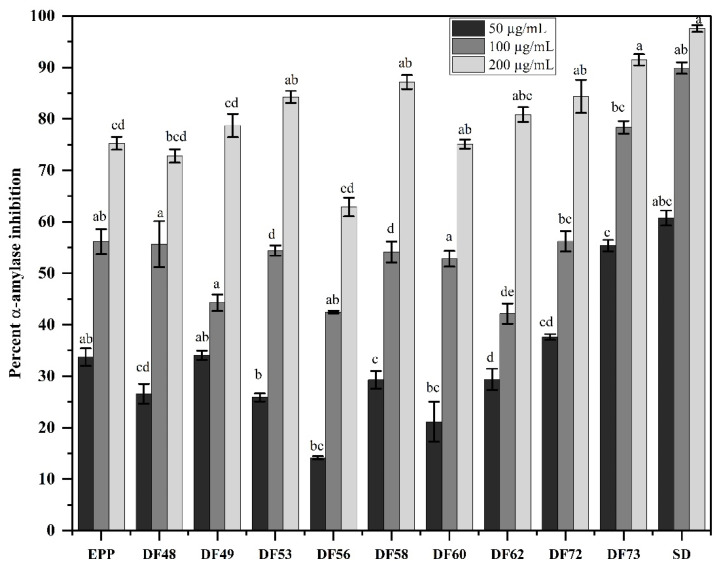
Percent inhibition of α-amylase activity at three different concentrations, where EPP: ethanol precipitated polysaccharides, DF48–DF73 oligosaccharide fractions obtained through microbial digestion of EPP, and SD: standard drug (acarbose). Data is represented as mean ± SD. Means represented with different letters (i.e., a, b, c ….) are significant from each other at *p* = 0.05.

Alpha-amylase (α-amylase) and alpha-glucosidase (α-glucosidase) are the two main carbohydrate-digesting enzymes accountable for playing a key role in regulating blood glucose levels. Both of these enzymes work in conjugation, where α-amylase initiates the breakdown of carbohydrates by catalyzing the hydrolysis of 1,4-glycosidic associations, while α-glucosidase converts disaccharides into monosaccharides, thus increasing blood glucose levels. Thus, the inhibitors of both of these enzymes help in decreasing postprandial BGL [32]. The oligosaccharides obtained from microbial digestion of EPP from cress seed mucilage showed promising α-amylase inhibitory potential as compared to acarbose (the standard drug), as portrayed in Figure 4. Among the screened fractions, DF73 exhibited strong potential by inhibiting the catalytic activity of α-amylase in a dose dependent manner. At 50 µg/mL concentration, DF73 inhibited the enzymatic activity by 55.38 ± 1.09%, which was increased to 78.33 ± 1.24% and 91.46 ± 1.09% at 100 µg/mL and 200 µg/mL, respectively. α-amylase inhibitors lower the rate of food starch digestion, which in turn avoids the abnormal increase in BGL after a meal. Consequently, screening for novel α-amylase inhibitors as hypoglycemic agents is still under research [33]. Earlier research reported that natural polysaccharides, such as *Fructus Mori* polysaccharides and *Momordica charantia* polysaccharides, markedly suppressed α-amylase activity [34]. Polysaccharides induce their α-amylase inhibitory effect through two potential mechanisms. Initially, polysaccharides inhibit the digestion of starch by α-amylase by adhering to the starch molecules, thereby impeding enzyme access to the substrate. Furthermore, some groups within polysaccharides, mainly carboxyl acid groups, interact with the amino acid residues in α-amylase through the formation of hydrogen bonds. This interaction results in the production of a polysaccharide/amylase complex, altering the spatial arrangement of the enzyme [35]. Uronic acid, a derivative of hexuronic acid, is frequently present in polysaccharides and has been demonstrated for its diverse biological effects, such as enzyme inhibition [36]. The high uronic acid content in DF73 indicates its contribution to the observed inhibitory effect against α-amylase.

### 2.6. In Vitro Alpha-Glucosidase Inhibition Potential of Oligosaccharide Fractions

**Figure 5:**
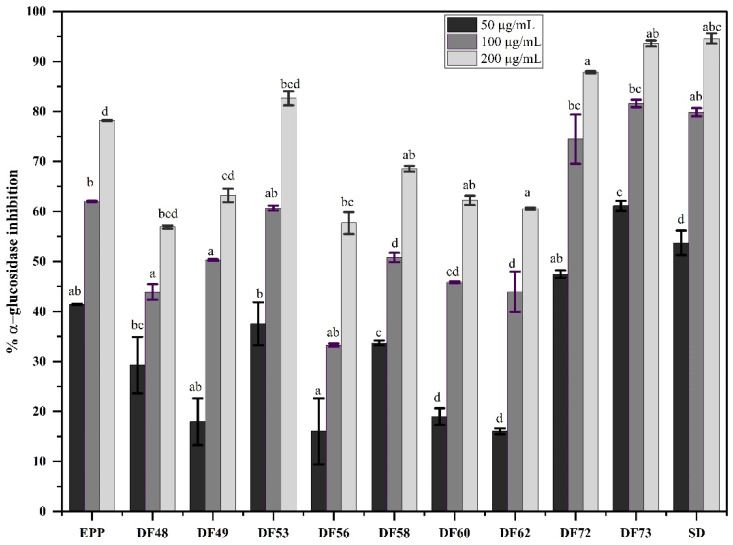
Percent inhibition of α-glucosidase activity at three different concentrations, where EPP: ethanol precipitated polysaccharides, DF48–DF73 oligosaccharides fraction obtained through microbial digestion of EPP, and SD: standard drug (acarbose). Data is represented as mean ± SD. Means represented with different letters (i.e., a, b, c ….) are significant from each other at *p* = 0.05.

Alpha-glucosidase is a membrane-bound carbohydrate-digesting enzyme found in the small intestine. Inhibition of α-glucosidase plays a significant role in the homeostasis of blood glucose levels. Acarbose, miglitol and voglibose are known α-glucosidase inhibitors that reduce and delay postprandial blood glucose levels [37]. In addition, several studies have reported that polysaccharides extracted from natural sources have excellent potential for α-glucosidase inhibition [38]. As illustrated in Figure 5, both the standard drug (acarbose) and oligosaccharide fractions of EPP exhibited strong α-glucosidase inhibitory potential in a concentration-dependent manner. The α-glucosidase inhibition potential of fraction DF73 was comparable to that of acarbose; at 200 µg/mL, the inhibition of DF73 was calculated to be 93.59 ± 0.56%, while that of acarbose was 94.56 ± 1.03%. Half-maximal inhibitor concentration (IC_50_) is usually used to determine the concentration of the inhibitors to inhibit the enzymatic activity by 50%. Table 2 shows the IC_50_ values of oligosaccharide fractions along with acarbose. The IC_50_ values of acarbose were 41.29 ± 3.12 µg/mL, while those of DF73 were 29.26 ± 2.68 µg/mL. Acarbose primarily inhibits the catalytic activity of α-glucosidase by holding the hydrophobic patch of the enzyme [38]. However, it can be assumed that oligosaccharide fractions may encapsulate the enzyme, substrate or both, thereby enhancing the inhibition rate [39]. The presence of α-(1, 4) glycosidic linkages is a vital constituent of the α-glucosidase inhibitory effect [40]. A previous study reported that polysaccharides extracted from *Gynostemma pentaphyllum* showed strong α-glucosidase inhibitory potential by inhibiting the catalytic activity of the enzyme by 63% at 3 mg/mL [41]. However, the strong α-glucosidase inhibitory activity of our samples might be due to the microbial digestion producing novel oligosaccharides with a different monosaccharide composition.

### 2.7. Hypoglycemic Effect of Oligosaccharide Fractions

**Figure 6:**
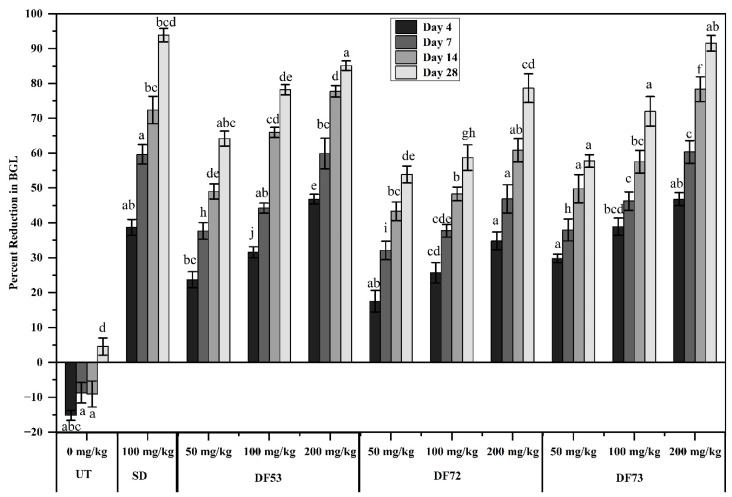
Percent reduction in blood glucose of STZ-induced diabetic mice, where UT: untreated diabetic control, SD: diabetic mice treated with 100 mg/kg of metformin, DF53–DF73 oligosaccharides fraction obtained through microbial digestion of EPP. Data is represented as mean ± SD. Means represented with different letters (i.e., a, b, c ….) are significant from each other at *p* = 0.05.

Streptozotocin (STZ) is a classical diabetogenic chemical that exerts selective alkylation of DNA in the cell death of the pancreas, thus reducing DNA synthesis and insulin production. STZ exposure to experimental animals causes destruction of the β-cells of the pancreas which leads to hyperglycemia, a condition similar to diabetes in humans [42]. Plant-derived mucilage stands out as an ideal choice for pharmaceutical applications because of several advantages, including being cost-effective, biocompatible, non-toxic, eco-friendly and readily modifiable. Biodegradability, abundant supply and easy processing further add to their benefits. The effect of oligosaccharide fractions on blood glucose levels (BGL) in STZ-induced diabetic mice over a 4-week period and the percent reduction in BGL are depicted in Figure 6. The initial BGL was monitored in both the STZ untreated group and the treated group, where the BGL was almost the same and within the error range. The BGL was monitored after days 4, 7, 14 and 28. Figure 6 indicates that a significant reduction was recorded in BGL in the mouse group treated with metformin and oligosaccharide fractions as compared to diabetic control group. The mean percent decrease in BGL in the mouse group treated with 200 mg/kg of DF73 was 46.79 ± 1.88% after day 4, 60.34 ± 3.25% after day 7, 78.35 ± 3.55% after day 14 and 91.5 ± 2.25% after day 28. Similarly, fractions DF53 and DF72 also reduced the BGL in dose- and time-dependent manner. After 28 days of treatment, at a 200 mg/kg concentration, an 85.10 ± 1.36% reduction in BGL was recorded for DF53 and 78.63 ± 4.12% for DF72. These values are comparable with metformin, which reduced the BGL by 93.86 ± 1.89% at 100 mg/kg after 28 days of treatment. Induction of STZ in mice induced visible features associated with diabetes, such as weight loss, excessive thirst (polydipsia) and excessive hunger (polyphagia). Besides, it also impedes glucose-induced-insulin secretion by inhibiting glucokinase, which is known as the glucose sensor of the β-cells of the pancreas [43]. Extracts from different medicinal plants have been found to have an insulinogenic effect as they activate pancreatic β-cells. The possible anti-hyperglycemic mechanism of these natural extracts is associated with either enhancing insulin release or regenerating pancreatic β-cells [44]. Consequently, several plants have been studied to induce anti-hyperglycemic effects by stimulating the production of insulin [45]. It can be assumed that oligosaccharide fractions reversed the situation, thereby reducing the BGL of STZ-induced diabetic mice. The antidiabetic activity of oligosaccharides involves several molecular mechanisms, including enhancing pancreatic function, inhibiting α-glucosidase, inhibiting insulin and leptin resistance, exerting anti-inflammatory effects, regulating gut microbiota and hormones and intervening with diabetic risk factors [46]. It is worth mentioning that no adverse effects were observed in experimental mice treated with different concentrations of oligosaccharides. 

### 2.8. Effect of Oligosaccharide Treatment on the Weight of STZ-Induced Diabetic Mice

**Figure 7:**
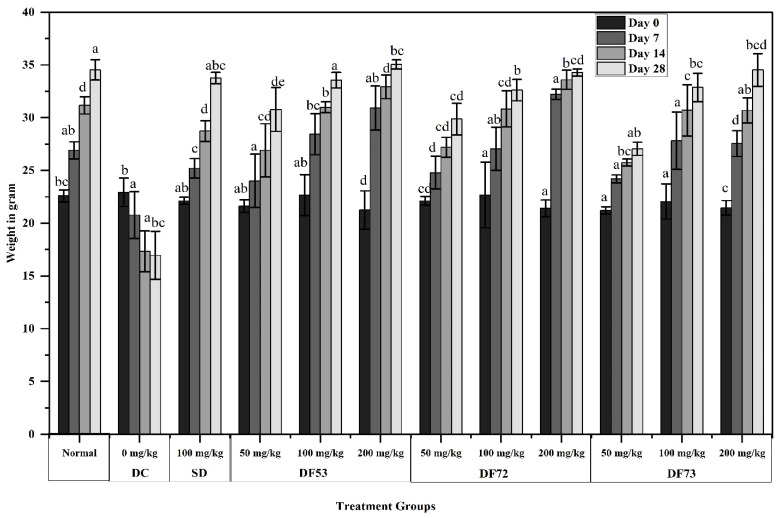
Effect on weight in STZ-induced diabetic mice, where DC: diabetic control, SD: diabetic mice treated with 100 mg/kg of metformin, DF53–DF73 oligosaccharides fraction obtained through microbial digestion of EPP. Data is represented as mean ± SD. Means represented with different letters (i.e., a, b, c ….) are significant from each other at *p* = 0.05.

Body weight loss is listed as one of the most common symptoms of diabetes, which may be due to the wasting of protein in situations where there is an unavailability of carbohydrates for utilization as an energy source [47]. Traditionally, management of diabetes is accompanied by weight gain. To study the effect of oligosaccharides from microbial digestion of EPP, eleven diabetic and one normal group were established. The average initial weight of each treatment group was measured, which was 22.59 ± 0.57 g for the normal group and 22.93 ± 1.36 g for the diabetic control group, as given in Figure 7. STZ-treatment induced a significant effect on the weight of untreated diabetic mice; on day 4, the weight was 22.93 ± 1.36 g and reduced to 16.95 ± 2.28 g after 28 days. The weight of the experimental mice was recorded every seven days, and the final weight was taken after 28 days. After 4 weeks of treatment, a significant increase was observed in the weight of diabetic mice treated with different oligosaccharide fractions and metformin, which served as the standard drug, while no increase was recorded in the diabetic control group. DF73 treatment increased the weight of diabetic mice from 21.45 ± 0.69 on day 4 to 34.52 ± 1.55 on day 28. Similarly, at 200 mg/kg, the weight of DF72-treated diabetic mice increased from 21.4 ± 0.79 to 34.2 ± 0.33. These results are similar to those of the positive control group, where metformin was applied as a standard drug. The results show that the weight of the mice in the diabetic control group were lost as compared to the control group (*p* < 0.05). The increase in the weight of mice treated with different concentrations of oligosaccharide fractions indicated that these fractions could effectively prevent body weight loss in mice with type 2 diabetes mellitus (T2DM) conditions.

### 2.9. Effect of Oligosaccharides Fraction on Blood Lipid Profile

**Figure 8:**
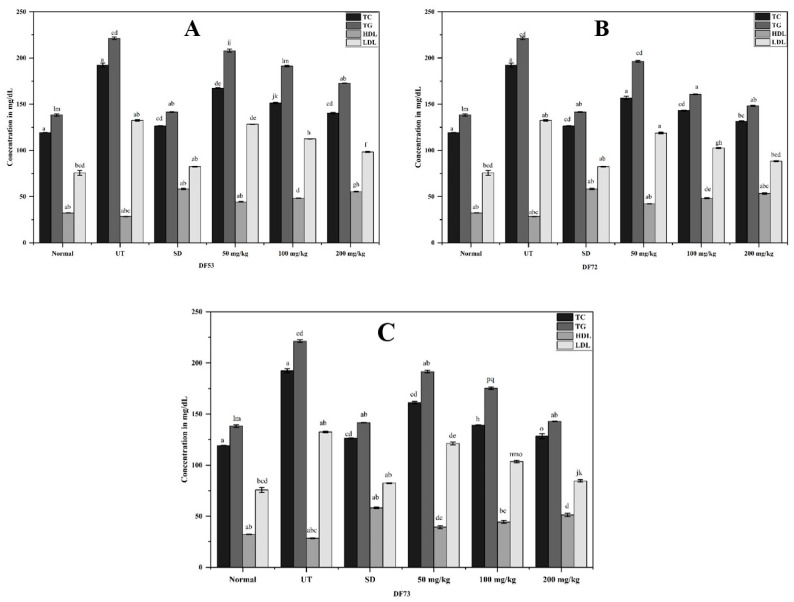
Effect on lipid profiles in STZ-induced diabetic mice, where DC: diabetic control, SD: diabetic mice treated with 100 mg/kg of metformin, where (**A**) DF53, (**B**) DF72 and (**C**) DF73, oligosaccharide fractions obtained through microbial digestion of EPP. Data is represented as mean ± SD. Means represented with different letters (i.e., a, b, c ….) are significant from each other at P=0.05.

The development of insulin deficiency and resistance significantly influences the enzymes and pathways involved in lipid metabolism, thereby inducing dyslipidemia, often occurring with T2DM. Dyslipidemia is often manifested as an enhanced level of triglycerides (TG), total cholesterol [48], low-density lipoproteins (LDL) and decreased levels of high-density lipoproteins (HDL) [49]. High levels of TG, TC, LDL-C and HDL-C contribute to the development of cardiovascular diseases in patients suffering from T2DM [50]. Thus, the development of novel bioactive ingredients aimed at the prevention of dyslipidemia will help in the effective management of T2DM. The blood lipid profiles of STZ-induced diabetic mice were assessed, and the results are portrayed in Figure 8. In the current study, STZ-induced chronic disorders in the blood lipid profile were observed while treatment with different oligosaccharide fractions resulted in a significant decrease in TC, TG and LDL-C levels. The levels of TC, TG and LDL-C were 192.34 ± 2.13 mg/dL, 221.27 ± 1.28 mg/dL and 132.45 ± 0.93 mg/dL, respectively, while they were reduced in the mouse group treated with 200 mg/kg of DF73, where the level of TC was 128.29 ± 2.39 mg/dL, TG was 142.89 ± 0.31 mg/dL and LDL-C was 84.51 ± 1.21 mg/dL. However, at low concentrations (50 mg/kg), none of the fractions induced any observable effect in STZ-treated mice. HDL-C, which is considered valuable cholesterol and lipoprotein, averts the tumbling of cholesterol by transporting cholesterol esters and endogenous cholesterol to the steroidogenic cells, thus preventing the onset of atherosclerosis [51,52]. Conversely, transportation of LDL-C from the liver to other tissues results in an elevated LDL-C level, which is considered the primary cause of cardiovascular diseases. The antidiabetic mechanism of *Abelmoschus esculentus* mucilage was shown to exert a significant reduction in TC, TG and LDL-C while enhancing the level of HDL-C in the serum profiles of diabetic mice [43]. *Apocynum venetum* polysaccharide extract showed a promising reduction in LDL-C and TG levels; however, no significant change was observed in the TC and HDL-C levels in alloxan-induced diabetic mice [53]. The hypolipidemic effect of polysaccharides has been determined to be associated with an increase in insulin secretion. Furthermore, enhanced insulin secretion has been found to increase cells’ ability to uptake blood glucose, inhibit the activity of hormone-sensitive lipase, and decrease the level of free fatty acids [54]. This further suggests that oligosaccharide fractions obtained from the microbial digestion of EPP could possibly regulate body lipid profiles, thereby promoting antidiabetic activity.

### 2.10. Effect of Oligosaccharide Fractions on Blood Serum Profiles

Diabetes mellitus is a metabolic disorder that adversely affects hepatocytes. Hepatocyte injury leads to the uncontrolled release of various intracellular substances directly into the blood stream. Measuring the concentration of hepatic enzymes in the main blood stream may provide valuable clues about hepatocyte malfunction [55]. Diabetes mellitus-associated liver damage is also responsible for causing elevated levels of SGOT and SGPT in systematic circulation. Both SGPT and SGOT are liver function enzymes [56]. STZ efficiently induces diabetes and causes a substantial increase in liver and kidney functions, as shown in Table 3. Treatment with oligosaccharide fractions obtained from microbial digestion of EPP effectively restored SGPT and SGOT levels after 28 days. Fraction DF73 induced a dose dependent effect on SGOT and reduced its level from 178.38 ± 0.92 U/L to 159.43 ± 1.27 U/L at 50 mg/kg, 96.28 ± 0.68 (U/L) at 100 mg/kg and 54.67 ± 0.57 (U/L) at 200 mg/kg. Similarly, at 200 mg/kg, DF73 reduced the SGPT level from 156.29 ± 2.56 (U/L) to 50.12 ± 1.35 (U/I) and the ALP level from 361.28 ± 0.19 (U/L) to 167.33 ± 1.23 (U/L). Our findings are in agreement with previous studies in the literature where leaf extract from *Wedelia chinensis* restored the level of these enzymes in the blood [49]. Similarly, polysaccharides extracted from *Acacia tortilis* and okra mucilage reduced the SGOT and SGPT levels in STZ-induced diabetic rats. These polysaccharides protect the liver either from the damage caused by diabetes or by STZ-subjection [57,58]. Creatinine is an entrenched indicator for evaluating the proper functioning of the kidneys, and that is why it is commonly measured in STZ-induced diabetic assays. In the current study, we also examined the effect of oligosaccharides on creatinine levels in SZT-induced diabetic mice. An increase in BGL can lead to kidney damage, impairing the organ’s ability to filter waste products, including creatinine, thus elevating its level in the blood [48]. It was found that oligosaccharide fractions promisingly reduced creatinine levels in the blood, indicating the protective effect of these polysaccharides in restoring kidney function.

### 2.11. Effect of Oligosaccharide Fractions on Liver Histology

**Figure 9:**
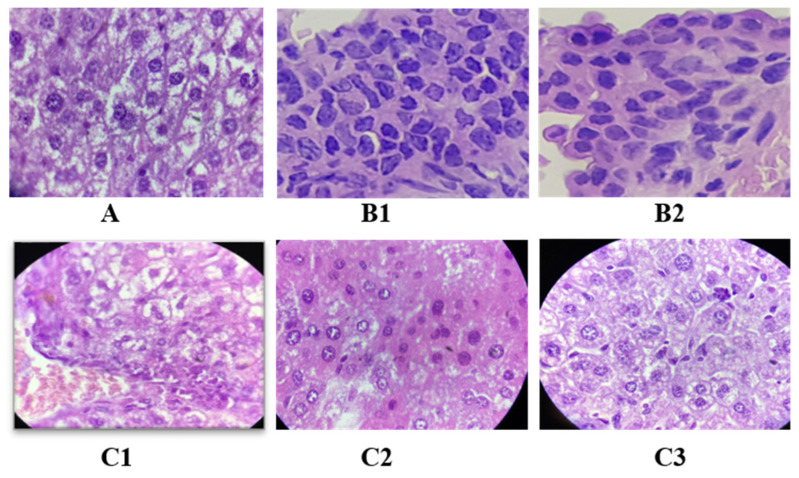

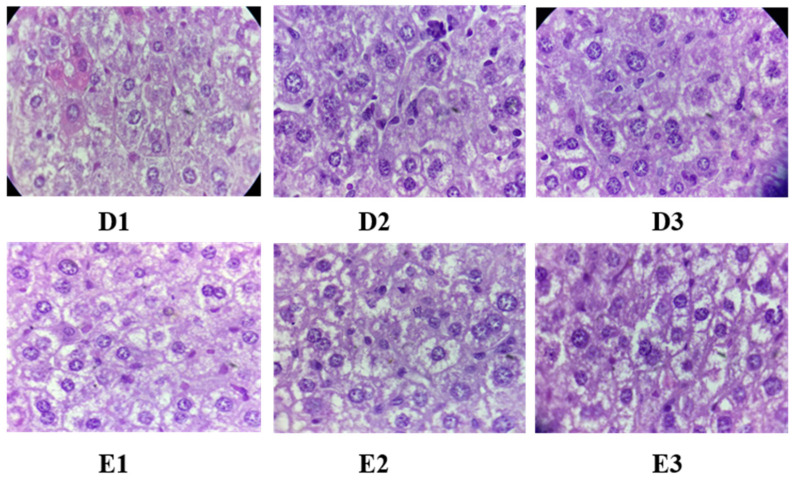
Effect on liver histology in STZ-induced diabetic mice under 40× objective lens of the light microscope, where A: diabetic mice treated with 100 mg/kg of metformin, B1 and B2: diabetic control, C1, C2, C3: diabetic mice treated with DF53, D1, D2, D3: diabetic mice treated with DF72 and E1, E2, E3: diabetic mice treated with DF73.

Persistent and abnormal hyperglycemia, along with oxidative stress, plays a crucial role in the pathogenicity of diabetes and its complications [59]. Histopathological observations of the H&E stained livers of experimental mice are illustrated in Figure 9. Figure 9 shows that diabetic mice treated with 100 mg/kg of metformin displayed a well-organized hepatic structure characterized by a neat arrangement of the hepatic cell cords and normal morphology. In contrast, experimental mice where diabetes was induced with STZ exhibited severe hepatocellular damage, including focal necrosis, inflammatory cell infiltration and mussy hepatic cords. Interestingly, the mouse group treated with 200 mg/kg of DF73 alleviated the disarray of the hepatic cords along with clear binuclear hepatocyte production, thus showing no liver abnormality compared to mice in the diabetic control group. STZ treatment caused significant damage to the liver by altering hepatocyte structures, causing hepatomegaly, dilation of hepatic sinusoid capillaries, apoptosis, increased adipogenesis and cell death in hepatocytes. Besides, STZ treatments also induced signs of inflammation in the liver, which needed to be reversed or stabilized in order to combat diabetes [60].

## 3. Materials and Methods

### 3.1. Chemicals and Reagents

All the chemicals used in this study were of HPLC grade. These included acetic acid, borax, citric acid, chlorobutanol, streptozotocin (STZ), dextrose, dextran, diclofenac sodium, ethanol, glucose, hydrochloric acid, iron chloride, methanol, m-hydroxy biphenyl, orcinol, sulfuric acid, thiobarbituric acid, sodium hydroxide, sodium chloride and sodium citrate from Sigma Aldrich (St. Louis, MO, USA); acetone and anthrone from Merck Millipore (Burlington, MA, USA); ascorbic acid from Omicron Sciences Limited (Hungerford, UK); Bio-Gel P-10 from Bio-Rad Laboratories (Hercules, CA, USA), hydrogen peroxide from BDH Laboratory Supplies (Dorset, UK); phenol from Ambion (Austin, TX, USA); phosphate-buffered saline from Oxoid (Basingstoke, UK); and sodium hypochlorite from DAEJUNG (Busan, Republic of Korea).

### 3.2. Polysaccharide Extraction

The mucilage was extracted by soaking the cress seeds in sterilized distilled water, in accordance with the procedure as previously described [61]. Briefly, 20 g of cress seeds were disinfected with 1% sodium hypochlorite (NaOCl) containing 0.67% active chlorine, soaked in 1000 mL of distilled water and left overnight at room temperature. The aqueous mixture was then filtered using a sterilized muslin cloth in order to isolate the mucilage. Long-chain polysaccharides were precipitated by treating cress seed mucilage with 75% (*v*/*v*) ethanol overnight on a shaker. Precipitated polysaccharides were collected by centrifugation at 4000× *g* rpm for 30 min. The supernatant was discarded, and the pellet containing polysaccharides was lyophilized. The dry weight of the polysaccharides was determined to be 4.67 ± 0.28 g. The obtained polysaccharides were confirmed using the phenol–sulfuric method as mentioned in Section 2.5 and stored at −20 °C for further use.

### 3.3. In Vitro Fermentation Using Yeast Strains

The free inoculum of the yeasts (*P. pastoris* and *P. kudriavzevii*) was cultured in YPD (yeast extract, peptone and dextrose) media for 24 to 48 h, and the cells were counted manually using a Neubauer chamber under a light microscope [62]. Further, 1 × 10^5^ yeast cells/mL were added to fermentation media containing 1% cress seed mucilage polysaccharides as a carbon source and 1 g/L, ammonium chloride (NH_4_Cl) as a nitrogen source. The fermentation reaction was incubated in a shaking incubator at 28–30 °C, and yeast growth was monitored for different time intervals, including 0, 6, 12, 24, 36, 48, 72 and 96 h. Once the reaction was completed, the yeast cell biomass was separated from the fermentation media using a centrifuge at 4500× *g* rpm for 25 min. The supernatant was collected in a fresh, sterilized tube, and the pellet was discarded.

### 3.4. Gel Permeation Chromatography (GPC)

The de-proteinated fermented product was freeze dried and fractionated by GPC. The de-proteination was performed by treating the fermented product with 10% trichloroacetic acid (TCA), followed by overnight incubation. After incubation, the sample was centrifuged at 5000× *g* rpm for 10 min. The freeze drying was carried out by freezing the fermented product at −30 °C and 100 Pa cavity pressure using a lab-scale freeze dryer (Yamato Scientific Co., Ltd., Santa Clara, CA, USA). The GPC column (2.8 cm width × 40 cm length) was packed with Bio Gel P-10, characterized by pore sizes ranging from 80 to 180 µm [63]. Briefly, the freeze-dried fermented product was re-dissolved at a concentration of 0.5% (*w*/*v*) and subjected to GPC at a ratio of 5% (*v*/*v*). Dextran served as the standard to calibrate the GPC column. Each fraction was collected in a 2 mL Eppendorf tube at a flow rate of 0.3 mL/min with deionized distilled water employed as the eluent. The collected fractions were then stored for further analysis. 

### 3.5. Biochemical Analysis

The total carbohydrate content in both ethanol precipitated polysaccharides [24] and size-based fractions collected through GPC was evaluated using the phenol–sulfuric acid assay [64]. Approximately 500 µL of sulfuric acid (98%) along with 10 µL of phenol (80%) were combined with 200 µL of oligosaccharide fractions. The resulting mixture was vortexed gently and allowed to incubate at room temperature for a duration of 20 min. The optical density of each fraction was measured at 490 nm, utilizing a UV-visible spectrophotometer. 

### 3.6. Fourier Transformed Infrared (FT-IR) Spectroscopy

The infrared (IR) spectra of the selected oligosaccharide fractions were obtained using an FT-IR spectrophotometer [65]. Selected oligosaccharide fractions were mixed and grounded with potassium bromide powder. The mixtures were pressed into a 1 mm pellet for the FT-IR measurement in the frequency range of 400 cm^−1^ to 4000 cm^−1^.

### 3.7. Gas Chromatography-Mass Spectrometry (GC-MS)

The monosaccharide composition of selected oligosaccharide fractions obtained through microbial digestion of EPP was quantified using gas chromatography-mass spectrometry (GC-MS) (GCMS-5977B Agilent Technologies, Santa Clara, CA, USA) as reported previously [66]. Known solutions of glucose, mannose, rhamnose, galactose, xylose and arabinose were used as external standards, while 2 mg of myo-inositol was used as an internal standard [67]. 

### 3.8. In Vitro α-Amylase Inhibition Assay

The in vitro antidiabetic potential of each oligosaccharide fractions was assessed following the method outlined by [68], with some modifications. Briefly, each fermented fraction obtained from GPC was added to a 96-well plate at three different concentrations (50, 100 and 200 µg/mL) along with 250 µL of α-amylase (0.5 mg/mL) (from bacterial source, EC: 3.2.2.1, catalogue: A0444-G) prepared in 0.02 N sodium phosphate buffer. The reaction was initiated by adding approximately 1% of 250 µL of starch prepared in 0.02 M sodium phosphate. The reaction plate was then left at room temperature for 10 min, terminated by the addition of dinitrosalicylic acid (DNS) and incubated in a hot water bath for 5 min. Upon the cooling of the reaction mix, the optical density of each fraction was calculated at 540 nm. Acarbose was utilized as a standard, and distilled water was employed as a blank. The percentage of α-amylase inhibition was determined using the standard formula as described by [69].

### 3.9. In Vitro α-Glucosidase Inhibition Assay

Cress seed mucilage polysaccharides and their fermented fractions were evaluated for their potential to inhibit α-glycosidase in vitro using a previously established protocol [68]. Briefly, 100 µL of α-glucosidase (α-glucosidase from *Saccharomyces cerevisiae*, GN5003-100UN, CAS: 9001-42-7, Sigma Aldrich) was pre-incubated with 50 µL of each saccharide fraction at three different concentrations. The reaction was initiated with the addition of 50 µL of 3.0 mM P-nitrophenyl-α-D-glucopyranoside, which was prepared in 20 mM phosphate buffer and incubated for 20 min. After incubation, the reaction was terminated by the addition of 0.1 M sodium carbonate (Na_2_CO_3_). The optical density of each saccharide fraction was calculated at 405 nm. Distilled water served as a blank, while acarbose was employed as standard. The percent α-glucosidase inhibition was determined using a standard formula described by [69].

### 3.10. In Vivo Antidiabetic Assay in Streptozotocin-Induced Diabetic Mice

#### 3.10.1. Induction of Diabetes

All the experiments performed in this study were approved and performed according to the guidelines provided by the ethical committee of the Department of Biotechnology, Abdul Wali Khan University Mardan (approval certificate number AWKUM/Biotech/2021/2615) and were in accordance with Good Laboratory Practices (GLP) of WHO. The male Balb/C mice were procured from the Veterinary Research Institute (VRI) in Peshawar and were allowed to acclimatize for one week at 25 ± 2 °C under a 12/12 h day/night cycle. Throughout this period, the mice had free access to food and water. After a one-week acclimatization period, the initial weight of the body was recorded, and the average weight of each group was kept within the same error range. Diabetes was induced in experimental mice through the intraperitoneal injection of freshly prepared 100 mg/kg of streptozotocin (STZ). The fasting blood glucose level (FBGL) was measured on day 4 (after 72 h) using an Accu-Chek Performa glucometer (Roche Diagnostics, Mannheim, Germany). Blood was drawn via the tail vein, and mice with FBGL greater than 11.1 mmol/L were categorized as diabetic.

#### 3.10.2. Experimental Design

The diabetic mice were grouped into different groups, with each group consisting of six mice. Group I served as a normal control, where the mice were administered distilled water; Group II served as a diabetic control, where diabetic mice were treated with vehicle only; Group III served as a positive control, where diabetic mice were treated with a standard drug dosage of 200 mg/kg. Groups IV–XII were administered with three different dosages of oligosaccharide fractions, including 50 mg/kg, 100 mg/kg and 200 mg/kg. All experimental mice received oral administration through gastric incubation once a day. Body weight and FBGL were recorded on days 7, 14 and 28. The decrease in FBGL was determined using the following standard equation:

$$Percent\;Reduction=\frac{\left(FBGL\;of\;diaetic\;control-FBGL\;of\;treated\;group\right)}{FBGL\;of\;diabetic\;control}*100$$

### 3.11. Blood Biochemical Analyses

After the completion of the in vivo experiment, mice were euthanized humanely, and blood was collected directly from the hearts of the experimental mice. The obtained blood was stored in an anti-coagulant-coated container for assessing further biochemical parameters [70]. The serum was screened for total cholesterol, total glycerides (TG), high-density lipids (HDL), low-density lipids (LDL), serum glutamic-oxaloacetic transaminase (SGOT), serum glutamic-pyruvic transaminase (SGPT), creatinine and alkaline phosphatase (ALP) using commercially available assay kits.

### 3.12. Histopathology of Mouse Livers

The livers of each experimental mouse was fixed in 10% formalin and embedded in paraffin wax [71]. 5 μm sections of the tissue were prepared carefully using a microtome, followed by hematoxylin and eosin (H&E) staining, and slides were photographed using a light microscope (Labomed TCM 400, Los Angeles, CA, USA).

### 3.13. Statistical Analysis

All experimental procedures were repeated three times for the sake of statistical analysis, and the results were expressed as means ± SD. Initial data were processed in Microsoft Excel (2013) and analyzed in GraphPad Prism 9.0 (GraphPad Software, Boston, MA, USA). Significant differences among the groups were determined using one-way analysis of variance (one-way ANOVA) using the Dunken *t*-test. A *p* value *p* ≤ 0.05 was considered significant. Graphs were made using Origin 2024. 

## 4. Conclusions

**Figure 10:**
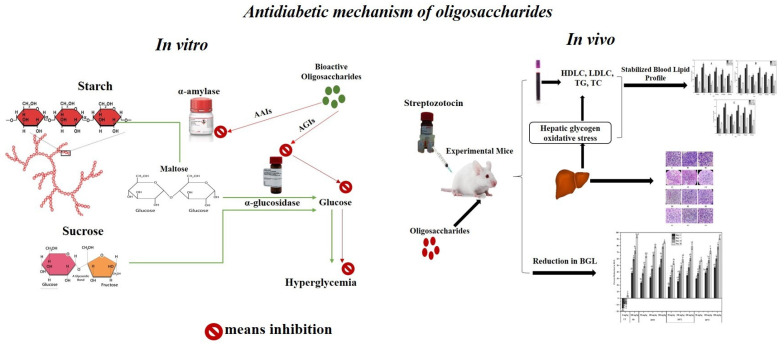
Antidiabetic mechanism of oligosaccharides obtained through the microbial digestion of cress seed mucilage polysaccharides.

This is the first study on the in vitro fermentation of ethanol-precipitated polysaccharides from cress seed mucilage for the production of antidiabetic oligosaccharides. Our study concludes that *P. pastoris* effectively utilized EPP as a carbon source and produced highly bioactive oligosaccharides, which significantly reversed the diabetogenic effect of STZ in diabetic mice (Figure 10). The GPC, utilizing Bio Gel P-10, effectively fractionated the fermented product and yielded several bioactive fractions. The biochemical characterization revealed the presence of various monosaccharides; however, it requires further structural characterization as the bioactivity of the oligo- and polysaccharides is often associated with their structural arrangement. The oligosaccharide fractions showed the potential to control body weight and normalize the BGL of the mice. The oligosaccharide fractions also had the tendency to modulate the lipid profile and liver and kidney functions, thereby preventing or mitigating the onset of possible cardiovascular diseases associated with diabetes. Moreover, our findings suggest possible mechanisms of action through enhanced glucose uptake, inhibition of carbohydrate-digesting enzymes and regulation of the various signaling pathways as shown in Figure 10. The observed antidiabetic effect of these oligosaccharide fractions offers exciting possibilities for their utilization in the development of complementary or alternative therapies for diabetes. Further research in this area has the potential to make a meaningful impact on the lives of individuals affected by diabetes and improve global health. Future research should optimize drug dosage, explore advanced delivery systems and elucidate the biological mechanisms of action of these oligosaccharides.

## Figures and Tables

**Table 1:** Percent monosaccharaide and disaccharide contents quantified through GC-MS in DF53, DF72 and DF73.

	Types of Monosaccharaides	DF53	DF72	DF73
Neutral Monosaccharides	Arabinose	11.67	4.53	3.12
	Fucose	4.56	2.09	1.96
	Xylose	8.73	2.13	Traces
	Galactose	13.27	5.21	2.06
	Glucose	21.21	11.67	8.61
	Rhamnose	-	4.03	5.61
	Sucrose	9.28	7.32	3.16
	Maltose	0.7	1.31	2.67
Acidic Monosaccharaides	Galacturonic Acid	26.34	54.58	67.38
	Glucuronic Acid	4.24	7.13	5.43

**Table 2:** Half maximal inhibitory concentration (IC_50_) of different enzymatic activities, where EPP: ethanol precipitated polysaccharides, DF48-DF73 oligosaccharides fraction obtained through microbial digestion of EPP, and SD: standard drug (acarbose).

	α-Amylase (IC_50_ in µg/mL)	α-Glucosidase (IC_50_ in µg/mL)
EPP	84.48 ± 2.86	67.19 ± 3.18
DF48	95.04 ± 1.98	139.48 ± 2.09
DF49	108.92 ± 4.21	120.88 ± 1.73
DF53	89.12 ± 3.09	72.87 ± 1.66
DF56	133.55 ± 2.17	161.13 ± 5.61
DF58	84.85 ± 1.88	96.04 ± 2.69
DF60	100.81 ± 0.9	127.81 ± 2.74
DF62	97.88 ± 2.12	135.76 ± 11.25
DF72	86.1 ± 2.64	50.47 ± 5.18
DF73	38.2 ± 1.12	29.26 ± 2.68
SD	29.18 ± 1.76	41.29 ± 3.12

**Table 3:** Effect on liver function in STZ-induced diabetic mice, where NC: normal control, DC: diabetic control, SD: diabetic mice treated with 100 mg/kg of metformin, DF53–DF73 oligosaccharide fractions obtained through microbial digestion of EPP. Data is represented as mean ± SD. Means represented with different letters (i.e., a, b, c ….) are significant from each other at *p* = 0.05.

Treatments	Doses (mg/kg)	SGOT (U/L)	SGPT (U/L)	ALP (U/L)	Creatinine (mg/dL)
NC	0.3 mL	65.63 ± 1.23 ab	47.85 ± 1.52 bc	165.45 ± 0.38 cd	1.23 ± 0.12 a
DC	0.3 mL	178.38 ± 0.92 a	156.29 ± 2.56 cd	361.28 ± 0.19 cd	3.60 ± 0.17 ab
SD	100	74.66 ± 0.57 abc	53.73 ± 1.83 a	160.57 ± 1.56 b	1.25 ± 0.09 c
DF53	50	158.75 ± 1.53 bc	147.26 ± 0.87 a	284.33 ± 0.93 a	3.12 ± 0.51 d
	100	119.27 ± 2.68 ab	119.41 ± 0.36 abc	221.46 ± 0.07 abc	2.73 ± 0.08 a
	200	78.28 ± 3.71 ab	73.54 ± 1.24 cd	182.55 ± 1.09 ab	2.08 ± 1.01 ab
DF72	50	165.23 ± 0.93 a	136.48 ± 3.21 ab	294.26 ± 1.28 bc	3.03 ± 0.19 bc
	100	112.52 ± 3.51 b	103.33 ± 1.27 a	272.58 ± 0.76 cd	2.03 ± 0.07 c
	200	71.89 ± 0.83 d	70.58 ± 0.97 c	202.39 ± 0.82 ab	1.55 ± 0.16 bcd
DF73	50	159.43 ± 1.27 cd	129.27 ± 0.58 bc	254.18 ± 1.08 a	2.43 ± 0.09 b
	100	96.28 ± 0.57 ab	94.56 ± 0.92 ab	199.92 ± 0.99 a	1.85 ± 0.15 cd
	200	54.67 ± 0.57 ab	50.12 ± 1.35 c	167.33 ± 1.23 a	1.29 ± 0.24 ab

## Data Availability

The original contributions presented in the study are included in the article, further inquiries can be directed to the corresponding authors.

## Abbreviations

ALP	Alkaline phosphatase
BGL	Blood glucose levels
DC	Diabetic control
DNS	dinitrosalicylic acid
EPP	Ethanol precipitated polysaccharide
FBGL	Fasting blood glucose level
FT-IR	Fourier transform infrared
GC-MS	Gas chromatography-mass spectrometry
GLP	Good Laboratory Practices
GPC	Gel permeation chromatography
H&E	Hematoxylin and eosin
HDL	High-density lipoproteins
HPLC	High-Performance Liquid Chromatography
IC_50_	Half-maximal inhibitor concentration
IR	Infrared spectroscopy
LDL	Low-density lipoproteins
NH_4_Cl	Ammonium chloride
PSA	Phenol–sulfuric acid
RBPS	Rice brain polysaccharides
SGOT	Serum glutamic-oxaloacetic transaminase
SGPT	Serum glutamic-pyruvic transaminase
STZ	Streptozotocin
T2DM	Type 2 diabetes mellitus
TC	Total cholesterol
TCC	Total carbohydrates content
TG	Total glycerides
UV	Ultraviolet
